# Commentary: Family caregivers and restless legs syndrome- sleep, quality of life and the need for family-centred nursing care

**DOI:** 10.1177/17449871261421137

**Published:** 2026-06-19

**Authors:** Fiona Tierney

**Affiliations:** Oxford Brookes University, UK

**Commentary to:** Everyday life experiences of family members of individuals with restless legs syndrome: a qualitative interview study (Odzakovic et al., 2026).

Family members often provide informal practical and emotional support for people with chronic conditions, and their relative’s illness can affect their own quality of life in many ways. However, family caregivers and healthcare professionals (HCPs) agree that their needs – practical, emotional and informational – can go unnoticed and unmet as the focus of care is on the individual living with illness (Jensen et al., 2021; Marín-Maicas et al., 2021). Nurses’ professional role includes providing care and services to a person’s family members, with the International Council of Nurses (ICN) recognising the need to inform, involve, advocate, and initiate care for individuals *and families* (ICN, 2021). This makes nurses ideally placed healthcare professionals to address these often unacknowledged and unsupported needs.

This study provides knowledge of family members’ experiences which can empower nurses in supporting families, as well as individuals with Restless Legs Syndrome (RLS). In their qualitative interview study, Odzakovic et al. (2026) present the experiences of family members of people with RLS, a widespread and often distressing sensory-motor disorder which causes pain and the urge to move the legs in sufferers (Broström et al., 2023, 2024). The views of 25 partners and adult children of people diagnosed with RLS – a large qualitative sample – were collected in telephone interviews and analysed by a team of four nurses experienced in treating RLS. Quality of life is reduced for individuals with this long-term illness, and, as Odzakovic et al. (2026) show can also be greatly impacted for their family members. Partners and adult children living with individuals with RLS face disruption to daily activities, socialising and difficult emotions around adjusting to changes to life. They also experience sleep disturbance and fatigue through supporting their family member with night-time discomfort caused by the condition.

RLS is classified as a movement-related sleep disorder (American Academy of Sleep Medicine, 2014), and there is growing recognition that a family systems approach to sleep is needed as an individual’s sleep impacts and is influenced by the family unit (e.g. Gunn and Eberhardt, 2019). Odzakovic et al. (2026) show that family members of individuals with RLS may be vulnerable to sleep disorders due to both nighttime caregiving and negative emotions about adjusting to life with their family member’s condition. They also illustrate how fatigue can affect family relationships and functioning on a daily basis. As evidence grows that sleep is essential for physical and mental health (e.g. Medic et al., 2017), understanding of the sleep challenges faced by family members is important so that they can be identified and addressed. Caregiving is physically and emotionally demanding and sleep is a priority in maintaining caregiver health and wellbeing and preventing burn-out. Nurses’ person-centred approach and role as advocates makes them ideally placed HCPs to discuss sleep challenges with family members and initiate referrals where appropriate.

Odzakovic et al. (2026) explore specific challenges to maintaining physical and emotional health for family members of people with RLS, along with their varied coping strategies. This offers nurses useful entry points for supportive conversations and information-sharing around areas which may not be routinely discussed, such as sexual health and establishing and maintaining social networks. Varied ways that families cope are presented, such as adapting social schedules and finding new activities to enjoy as a family, along with the importance of family members finding ways to prioritise themselves at times. As trusted HCPs, nurses can give permission to family members to feel that they matter too.

Odzakovic et al. (2026) argue for policymakers to prioritise the views and needs of family members by including in clinical guidelines a Family Systems Nursing approach (Bell, 2009), which recognises that families actively participate in adapting to an individual’s chronic illness and that their health and well-being are also affected. Shared decision-making, involving communication between HCPs and patients about their needs and wishes, is recommended to bring about the best outcomes in RLS (Björk et al., 2024) and other conditions. Odzakovic et al. (2026) advocate for expanding decision-making to include family members, who are impacted by and assist in managing the condition. This could benefit the emotional health of family members, who need their realities to be understood, recognised, and validated and to have access to appropriate support where required. To facilitate shared decision-making, education and information for both families and nurses on how best to communicate and work together over the course of care are needed. With such training, nurses’ skills in advocacy and delivering person-centred, holistic care means they are the ideal profession to lead in improving care for families affected by RLS.
